# Assessment of cerebrovascular reserve with N-isopropyl-p-[^123^I]-iodoamphetamine time series analysis in patients with cerebrovascular disease

**DOI:** 10.1097/MD.0000000000025617

**Published:** 2021-04-23

**Authors:** Kiyohide Kakuta, Kenichiro Asano, Kosuke Katayama, Hiroki Ohkuma

**Affiliations:** Department of Neurosurgery, Hirosaki University Graduate School of Medicine Zaifu-cho 5, Hirosaki-shi, Aomori, Japan.

**Keywords:** cerebral blood flow, cerebrovascular reserve, redistribution phenomenon, single-photon emission tomography

## Abstract

Using N-isopropyl-p-[^123^I]-iodoamphetamine(^123^I-IMP) and single-photon emission computed tomography (SPECT), the relationship between cerebrovascular reserve and the ^123^I-IMP redistribution phenomenon was investigated.

The 50 patients who matched the inclusion criteria were divided into control and ischemia groups, and the redistribution phenomenon was examined on resting images. The delayed images showed higher ^123^I-IMP accumulation in lesions in the middle cerebral artery(MCA) area and anterior cerebral artery(ACA) area, these watershed areas in the ischemia group than in the control group, confirming that the redistribution phenomenon exists with statistical significance (Wilcoxon test; control group vs ischemic group in the ACA area[*P* = .002], ACA-MCA watershed area(*P* = .014), MCA area(*P* = .025), and MCA-posterior cerebral artery(PCA) watershed area(*P* = .002). The patients were then divided into 4 types according to the Kuroda grading system, and the difference in the redistribution phenomenon was investigated between type III and the other 3 types.

Compared with type I and type II, type III had a significantly lower rate of decrease in the radioisotope (RI) count, verifying the redistribution phenomenon (Student *t* test: type I vs type III in the ACA area(*P* = .008), ACA-MCA watershed area(*P* = .009), MCA area(*P* < .001), and MCA-PCA watershed area(*P* = .002); type II vs type III in the ACA area(*P* = .004), ACA-MCA watershed area(*P* = .2575), MCA area(*P* < .001), and MCA-PCA watershed area(*P* < .001). No significant difference between type III and type IV was observed in any area [(Student *t* test: type III vs type IV in the ACA area(*P* = .07), ACA-MCA watershed area(*P* = .38), MCA area(*P* = .05), and MCA-PCA watershed area(*P* = .24)].

The redistribution phenomenon is associated with resting cerebral blood flow (CBF), but not necessarily with cerebral vascular reactivity (CVR).

## 1 Introduction

The tracer N-isopropyl-p-[^123^I] iodoamphetamine (^123^I-IMP), developed by Winchell et al,^[1]^ accumulates relatively quickly and is stored in the brain parenchyma via free diffusion through the blood brain barrier. Previous studies have applied ^123^I-IMP to single-photon emission computed tomography (SPECT) of central nervous system disorders such as cerebrovascular disorders.^[2]^

Although various hypotheses have been posited regarding the mechanism of accumulation, distribution, and elimination of this tracer in brain tissue, many points remain unclear. It has been reported that delayed images can show the redistribution phenomenon of ^123^I-IMP in reversible ischemic lesions in brain tissue.^[3]^ However, its mechanism has yet to be established and the technology has not been applied in the clinical setting.

In ^123^I-IMP SPECT, evaluation of cerebrovascular reserve by acetazolamide challenge is inversely correlated with a risk of cerebral infarction.^[4]^ This test procedure is widely used in clinical practice but it is known to occasionally induce serious side effects such as pulmonary edema.^[5]^

Several investigators have demonstrated that cerebrovascular reserve is inversely correlated with oxygen extraction fraction. Misery perfusion, which represents potentially viable tissue,^[6]^ can be identified by demonstrating an increased oxygen extraction fraction.^[7]^ However, few studies have systematically investigated the relation between the redistribution phenomenon of ^123^I-IMP and cerebrovascular reserve.

In the present study, early and delayed ^123^I-IMP images were examined, and the feasibility of assessing cerebrovascular reserve by quantitatively evaluating the redistribution phenomenon observed in the delayed images as a substitute for the acetazolamide challenge test was determined.

## 2 Materials and methods

### 2.1 Patient selection

**Figure 1:**
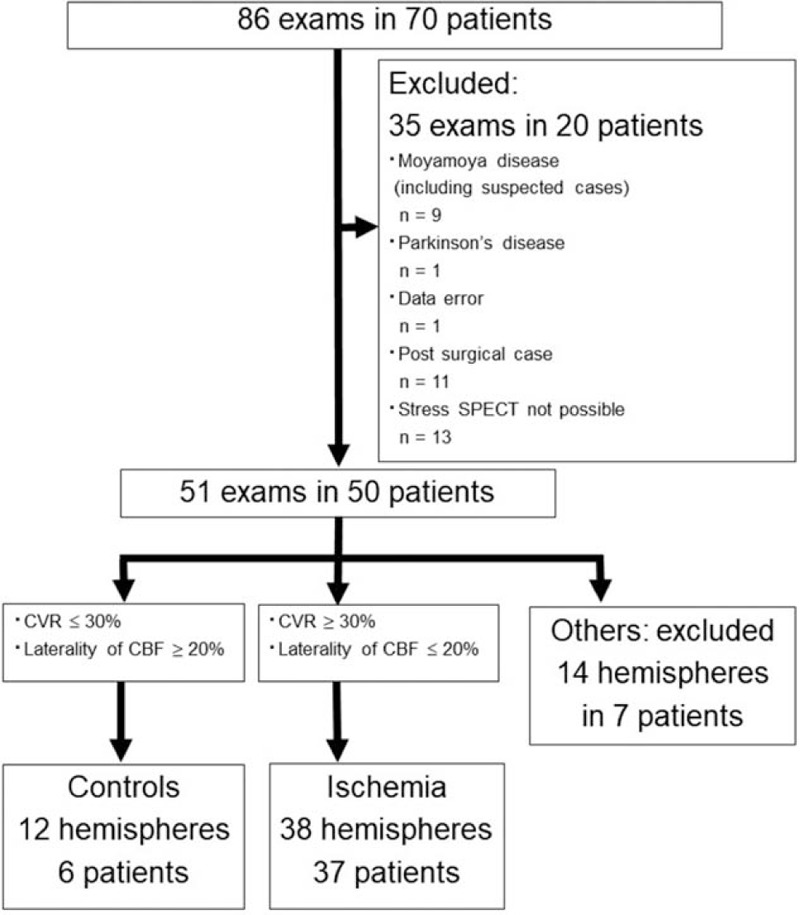
Trial profile of all patients. The CONSORT chart outlines the selection of patients who were included in our retrospective analysis.

A total of 86 examinations were performed in 70 patients with a main cerebral artery stenosis/occlusion requiring cerebrovascular reserve evaluation between June 2013 and July 2017. There were 60 men and 26 women, with a median age of 62 years. Included in the study were patients without infarction within the evaluated hemisphere, without a history of surgery or endovascular treatment, and without a brain disorder other than Moyamoya disease or an ischemic lesion. A final total of 51 examinations in 50 patients who met these criteria were analyzed. Those with good cerebrovascular reserve, with acetazolamide cerebral vascular reactivity (CVR) ≥30%, and no bilateral difference or infarct lesions were assigned to the control group (6 examinations in 6 patients, 12 hemispheres). Those with decreased CVR (≤30%), with bilateral difference on the images but without infarct lesions in these areas were assigned to the ischemia group (38 hemisphere examinations in total in 37 patients: 23 left hemisphere examinations in 22 patients, and 15 right hemisphere examinations in 15 patients). The remaining 7 examinations in 7 patients were excluded from the analysis (Fig. 1, Table 1).

**Table 1:** Background and clinical characteristics of patients.

	Total	Controls	ACA	ACA-MCA	MCA	MCA-PCA
N	43	6	11	27	27	31
Age(yr), (median [IQR]), range	62 (57.25–67.25), 34–80	66 (54.75–66), 47–75	68 (61–74.75), 34–80	63 (60.5–67.75), 34–80	61 (52.5–68.75), 34–80	62 (58–68.5), 34–80
Sex (M: F)	33: 10	4: 2	11: 0	21: 6	20: 7	25: 6
Diagnosis						
ICA stenosis (Rt: Lt)	0: 5	0: 1	0: 1	0: 2	0: 3	0: 4
ICA occlusion (Rt: Lt)	8: 10	2: 1	5: 4	5: 7	2: 6	5: 8
MCA stenosis (Rt: Lt)	5: 5	1: 0	0: 0	3: 3	4: 5	3: 5
MCA occlusion (Rt: Lt)	5: 5	1: 0	0: 1	4: 3	3: 4	3: 3

### 2.2 Study procedures

Imaging was performed using an Infinia Hawkeye 4 SPECT/CT system (General Electric Company, Boston, MA) with a matrix size of 128 mm × 128 mm, and an ELEGP step & shot collimator. ^123^I-IMP: Iofetamin 222 MBq was used as the nuclide. Resting SPECT examination and acetazolamide challenge SPECT examination were performed with the 2-day method. In resting SPECT, the nuclide was administered intravenously with the patient in a resting position with the eyes closed, and arterial blood was collected 10 minutes after administration. Early and delayed images were obtained at 20 to 40 minutes and 170 to 190 minutes after administration, respectively. The acetazolamide challenge SPECT examination was performed after an interval of at least 1 week. Five minutes after nuclide administration, 1000 mg of acetazolamide were administered intravenously. Arterial blood was collected 10 minutes after administration, and images were obtained 20 to 40 minutes after administration.

Three-dimensional stereotactic region of interest (ROI) template software was used for analysis of cerebral blood flow (CBF). The CBF images were automatically partitioned into defined regions to avoid variability in manual ROI setting. In early and delayed imaging of resting SPECT and acetazolamide stress SPECT, the radioisotope (RI) count captured with the gamma camera and the CBF value were calculated and analyzed.

**Figure 2:**
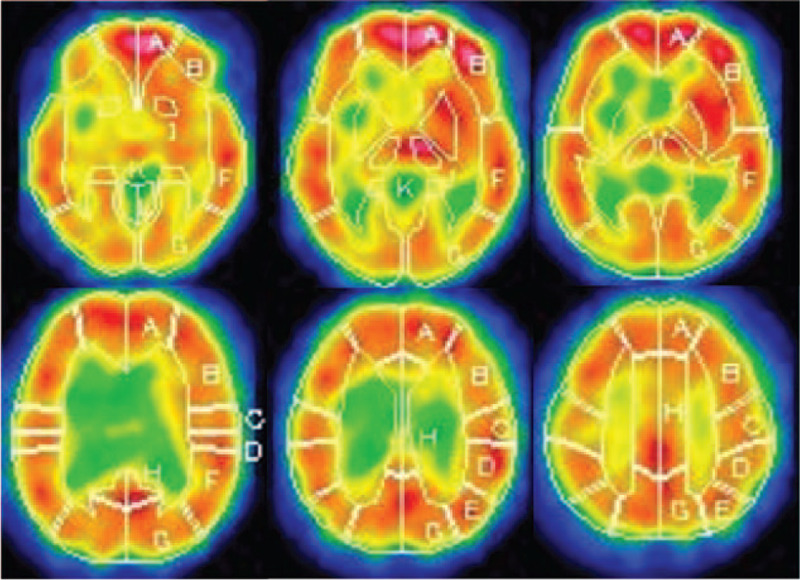
Three-dimensional stereotactic region-of-interest (ROI) template. The CBF values and RI counts of 318 ROIs are calculated automatically. Four ROIs are selected for use in the present study (A = ACA area, B = ACA-MCA watershed area, F = MCA area, E = MCA-PCA watershed area).

In examination 1, the anterior cerebral artery (ACA) area, ACA-middle cerebral artery (MCA) watershed area, MCA area, and MCA-posterior cerebral artery (PCA) area were compared between the control group and the ischemia group. ROIs were set on the three-dimensional stereotactic region of interest templates as follow: ACA area as A, ACA-MCA watershed area as B, MCA area as F, and MCA-PCA watershed area as E (Fig. 2). When there were multiple applicable ischemic areas in the same patient, each ischemic area was analyzed separately. In early and delayed imaging of resting SPECT, the RI count captured by the gamma camera was calculated for each area within the ROI. Typically, the RI count decreases from early to delayed imaging; however, because IMP sweep is slower at sites with decreased blood flow, a further decrease in the RI count rate occurs with the decrease from early to delayed imaging, and the RI count can increase. This is observed on the image as the redistribution phenomenon, which increases from early to delayed imaging.

The rate of decrease in the RI count is calculated using the following equation:

$$\text{Rate}\;\text{of}\;\text{decrease}\;\text{in}\;\text{the}\;\text{RI}\;\text{count}=\frac{\text{early}\;\text{SPECT}\;\text{RI}\;\text{count}\backslash -\text{delay}\;\text{SPECT}\;\text{RI}\;\text{count}}{\text{early}\;\text{SPECT}\;\text{RI}\;\text{count}}\times 100$$

In examination 2, the rates of decrease in the RI count according to the Kuroda grading system^[8]^ were compared in the aforementioned 4 areas. In the Kuroda grading system, each area is classified into 1 of 4 different types based on the acetazolamide stress CVR and resting CBF.

CVR is calculated using the following equation.^[9]^

$$\text{CVR}(\%)=\frac{\text{stress}\;\text{CBF}-\text{rest}\;\text{CBF}}{\text{rest}\;\text{CBF}}\times 100$$

The 4 classifications of the Kuroda grading system are as follows:

1. Type I. Resting CBF ≥34 ml/100 g/min and CVR ≥23%
2. Type II. Resting CBF ≥34 ml/100 g/min and CVR <23%
3. Type III. Resting CBF <34 ml/100 g/min and CVR <23%
4. Type IV. Resting CBF <34 ml/100 g/min and CVR ≥23%

Among these 4 classifications, the RI count decrease rate of type III, which corresponds to Powers classification stage 2, was compared with that of the other types, and significant differences were identified.

This study was approved by the ethics committee of Hirosaki University Graduate School of Medicine our institution, and SPECT data were collected during the process of medical treatment of patients who received an explanation about the study and gave their consent to participate. Data were anonymized and handled following our hospital's regulations on privacy protection.

### 2.3 Statistical analysis

The statistical analyses were performed using JMP13 (SAS Institute Inc., Cary, NC) on a computer with the Windows 7 operating system.

In examination 1, the Wilcoxon test was used to determine whether the ischemia group had a significantly lower rate of decrease in the RI count compared with the control group for each area. In examination 2, Student *t* test was used to determine whether type III has a significantly lower rate of decrease in the RI count compared with the other types for each area in examination 2. A *P* value of less than .05 was considered significant.

## 3 Results

### 3.1 Examination 1

**Figure 3:**
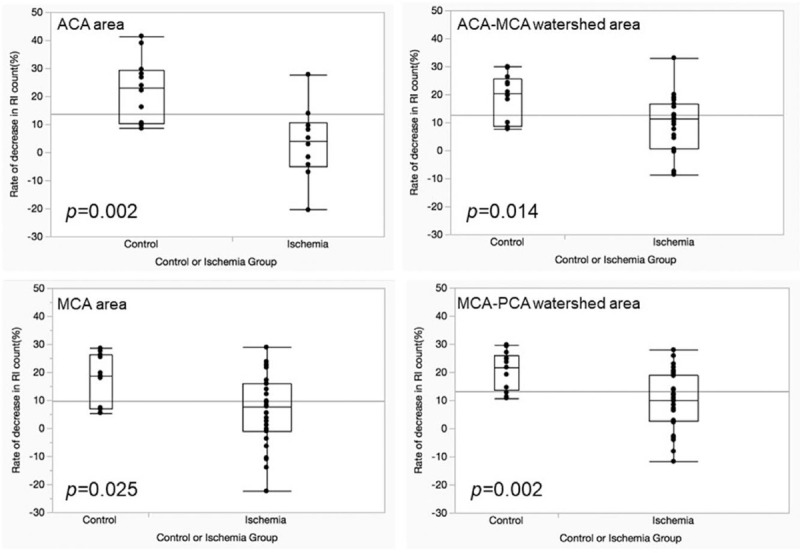
Comparison between the control and ischemic groups of the rate of decrease in the RI count. There are significant differences between the control and ischemic groups in all 4 areas.

The comparisons of the rates of the decreases in the RI count in the delayed images in each area between the control group and ischemia group showed significant differences (*P* = .002 for ACA area, *P* = .014 for ACA-MCA watershed, *P* = .025 for MCA area, and *P* = .002 for MCA-PCA watershed). These results suggest the presence of the redistribution phenomenon in the ischemia group (Fig. 3).

### 3.2 Examination 2

The correlation between the Kuroda grading system and the rate of decrease in the RI count was examined. In particular, the difference was analyzed in terms of Kuroda grading type III, which corresponds to the clinically important Powers classification stage 2.

Type I was significantly different from type III in all 4 of these areas (*P* = .008 for ACA area, *P* = .009 for ACA-MCA watershed, *P* < .001 for MCA area, and *P* = .002 for MCA-PCA watershed).

Type II was significantly different from type III in 3 areas (*P* = .004 for ACA area, *P* = .2575 for ACA-MCA watershed, *P* < .001 for MCA area, and *P* *<* .001 for MCA-PCA watershed).

However, in all 4 of these areas, no significant difference was observed between types III and IV (*P* = .07 for ACA area, *P* = .38 for ACA-MCA watershed, *P* = .05 for MCA area, and *P* = .24 for MCA-PCA watershed).

**Figure 4:**
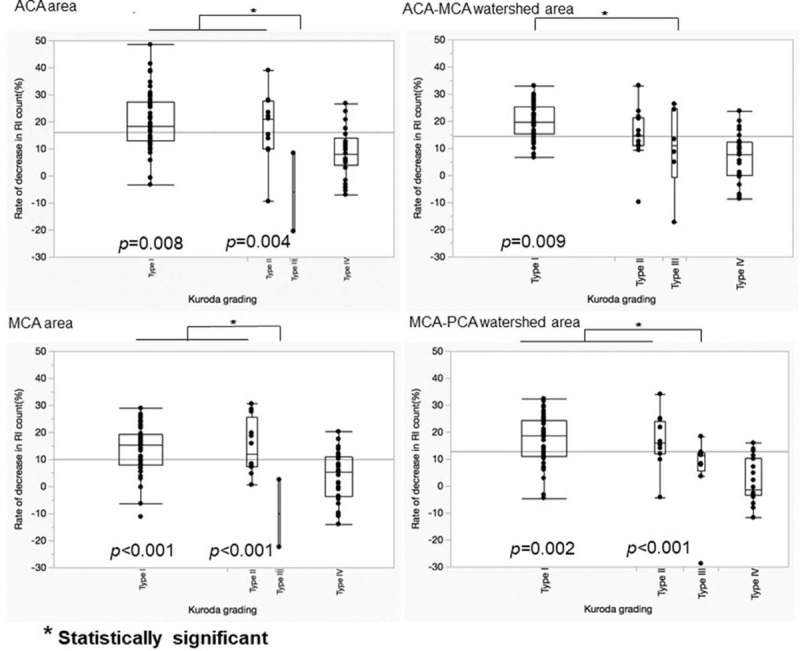
Comparison between type III and the other 3 types. In the all 4 of these areas, type I is significantly different from type III. Type II is also different from type III in 3 areas (ACA area, MCA area, MCA-PCA watershed area), but there are no significant differences between type III and type IV in all 4 areas.

These results indicate that Kuroda grading type III had a significantly lower rate of decrease in the RI count compared with types I and II, demonstrating a significant redistribution phenomenon in type III. In contrast, types III and IV were not significantly different, indicating that type IV exhibits a similar redistribution phenomenon to type III (Fig. 4).

## 4 Discussion

N-isopropyl-p[^123^I] IMP was developed as a local CBF tracer for SPECT. ^123^I-IMP is fat-soluble with a high migration rate from the blood into the brain. The first- pass extraction is reported to be 90%^[10]^ or 92%,^[2]^ and washout from the brain tissue is slow. Because ^123^I-IMP is retained for a long time, it is therefore suitable for SPECT. The early images reflect the local CBF and are widely used in clinical practice.

The redistribution phenomenon is observed in ^123^I-IMP delayed images, although its mechanism and clinical significance remain largely unknown.^[11]^ However, since its presence in sites with decreased cerebrovascular reserve indicates the potential of infarction and misery perfusion areas, it has been suggested for use as an index of brain tissue viability.^[3]^

Misery perfusion is characterized by mildly decreased blood flow and is a recoverable condition in which brain function and metabolism are maintained.^[12]^ Misery perfusion can be identified by demonstrating an increased oxygen extraction fraction which is inversely correlated with cerebrovascular reserve.^[7]^

Treatment of main cerebral artery stenosis or occlusion involves the identification of such misery perfusion followed by revascularization. Cerebrovascular reserve evaluation using acetazolamide challenge may sometimes be necessary for diagnosis, but it can induce serious side effects.^[8]^

The redistribution phenomenon in the delayed image can be interpreted in many ways. The 2 main interpretations are:

1. redistribution is the process in which the non-specific binding of IMP in the brain tissue after early distribution reaches an equilibrium state in the delayed image ^[11]^; and
2. redistribution is the mathematical function of wash-in and wash-out of IMP in the brain tissue.^[13]^

The first element reflects brain metabolism whereas the second element reflects brain circulation, and it is thought that redistribution is observed as a composite of these elements. Redistribution itself is caused by brain circulation and metabolism, and the phenomenon is not directly linked to cerebrovascular reserve itself. However, SPECT evaluations based on the Powers classification are widely used clinically; in that classification system, augmented oxygen metabolism is observed in disease states of stage 2 or higher. Moreover, after main cerebral artery occlusion, spikes, or sharp waves are observed on electroencephalography around the ischemic lesion or at the watershed area, occasionally leading to convulsions.^[14]^ In this situation, brain metabolism, including anaerobic metabolism, is increased. Thus, brain circulation and brain metabolism are indirectly but closely associated with each other.

Based on such observations, this study investigated whether there is a correlation between these 2 factors. Specifically, Kuroda grading was used to examine the correlation between the decrease rate reflecting the ^123^I-IMP redistribution phenomenon and the CBF increase rate with acetazolamide. The results showed that the redistribution phenomenon was uncommon in Kuroda grading types I and II, but frequent in types III and IV. In Kuroda grading, types I/II and III/IV are distinguished based on resting CBF. Types III and IV have comparable redistribution, but they are distinguished based on CVR. The present results showed that the redistribution phenomenon is correlated with resting CBF, but its association with CVR could not be demonstrated.

Clinically, Kuroda grading type III corresponds to Powers classification stage 2 with misery perfusion.^[9]^ Although there is no corresponding Powers classification for type IV, such an area shows decreased nerve cell density and decreased CBF due to lower metabolic demand.^[15]^ To verify the difference between these 2 types, it may be necessary to conduct additional studies using specific conditions that reflect the metabolic state.

Although ^123^I-IMP degrades into water-soluble substances such as iodobenzoic acid,^[16]^ in cerebral infarct lesions with a disrupted BBB, these metabolic degradation products behave differently in the brain compared with fat-soluble IMP, and there is accumulation of these products regardless of tissue viability. Moreover, it has been indicated that tissue pH increases depending on the extent of ischemic tissue damage,^[17]^ and it has been reported that the pharmacokinetics of tissue IMP and its metabolites are consequently affected.^[18]^ IMP binds to relatively nonspecific amine receptors localized in the brain and cerebral capillary intima, but these receptors are affected by the extent of tissue damage as well as individual differences.^[19]^

The metabolic factors that affect such IMP accumulation include impermeable metabolite behavior, pH gradient, and receptor binding, which suggests that these elements regulate the redistribution phenomenon.

In other words, to evaluate cerebrovascular reserve using the redistribution phenomenon, it is necessary to perform further investigations taking into account these metabolic factors.

Benzodiazepine receptor binding potential (BRBP) on ^123^I-iomazenil SPECT imaging correlates with the cerebral metabolic rate of oxygen on positron emission tomography images in patients with ischemic cerebrovascular disease.^[20]^ The accuracy of BRBP/CBF asymmetry on SPECT is equivalent to that of the combination of CBF and cerebrovascular reserve to acetazolamide on SPECT for detection of misery perfusion in inpatients with unilateral major cerebral artery occlusive disease.^[21]^ These studies provide a possible new direction by comparing BRBP on iomazenil and the redistribution phenomenon in IMP delayed images.

## 5 Conclusions

A significant difference in the rate of decrease in the RI count was observed between the control group and the ischemia group, and observation of the redistribution phenomenon was confirmed, particularly in the watershed areas. Investigation of the redistribution phenomenon by the Kuroda grading system showed significantly greater redistribution in type III showing misery perfusion compared with type I and type II, but not with type IV.

Thus, to evaluate cerebrovascular reserve based on the extent of the redistribution phenomenon, it is necessary to assess metabolic factors, at the very least, as well as CBF.

## Acknowledgments

The authors gratefully acknowledge the work of past and present members of their department.

## Author contributions

**Conceptualization:** Kenichiro Asano.

**Formal analysis:** Kiyohide Kakuta.

**Project administration:** Kenichiro Asano.

**Supervision:** Kenichiro Asano, Kosuke Katayama, Hiroki Ohkuma.

**Validation:** Kiyohide Kakuta, Kenichiro Asano, Kosuke Katayama, Hiroki Ohkuma.

**Writing – original draft:** Kiyohide Kakuta.

**Writing – review & editing:** Kiyohide Kakuta.
